# Long-term remote organ consequences following acute kidney injury

**DOI:** 10.1186/s13054-015-1149-5

**Published:** 2015-12-28

**Authors:** Chih-Chung Shiao, Pei-Chen Wu, Tao-Min Huang, Tai-Shuan Lai, Wei-Shun Yang, Che-Hsiung Wu, Chun-Fu Lai, Vin-Cent Wu, Tzong-Shinn Chu, Kwan-Dun Wu, on behalf of the National Taiwan University Hospital Study Group on Acute Renal Failure (NSARF) and the Taiwan Consortium for Acute Kidney Injury and Renal Diseases (CAKs)

**Affiliations:** Division of Nephrology, Department of Internal Medicine, Saint Mary’s Hospital Luodong, 160 Chong-Cheng South Road, Luodong, Yilan 265 Taiwan; Saint Mary’s Medicine, Nursing and Management College, 160 Chong-Cheng South Road, Luodong, Yilan 265 Taiwan; Division of Nephrology, Department of Internal Medicine, MacKay Memorial Hospital, 92, Sec. 2, Zhongshan N. Road, Taipei, 10449 Taiwan; Division of Nephrology, Department of Internal Medicine, National Taiwan University Hospital Yun-Lin Branch, 579, Sec. 2, Yunlin Road, Douliu City, Yunlin County 640 Taiwan; Department of Internal Medicine, National Taiwan University Hospital, Bei-Hu Branch, 87 Neijiang Street, Taipei, 108 Taiwan; Division of Nephrology, Department of Internal Medicine, National Taiwan University Hospital, Hisn-Chu Branch, No.25, Lane 442, Sec. 1, Jingguo Road, Hsin-Chu City, 300 Taiwan; Division of Nephrology, Taipei Tzu Chi Hospital, Buddhist Tzu Chi Medical Foundation, Taipei, Taiwan; School of Medicine, Tzu Chi University, Hualien, Taiwan; Division of Nephrology, Department of Internal Medicine, National Taiwan University Hospital, 7 Chung-Shan South Road, Zhong-Zheng District, Taipei, 100 Taiwan

**Keywords:** Acute kidney injury, Renal replacement therapy, Organ crosstalk, Remote organ consequences

## Abstract

Acute kidney injury (AKI) has been a global health epidemic problem with soaring incidence, increased long-term risks for multiple comorbidities and mortality, as well as elevated medical costs. Despite the improvement of patient outcomes following the advancements in preventive and therapeutic strategies, the mortality rates among critically ill patients with AKI remain as high as 40–60 %. The distant organ injury, a direct consequence of deleterious systemic effects, following AKI is an important explanation for this phenomenon. To date, most evidence of remote organ injury in AKI is obtained from animal models. Whereas the observations in humans are from a limited number of participants in a relatively short follow-up period, or just focusing on the cytokine levels rather than clinical solid outcomes. The remote organ injury is caused with four underlying mechanisms: (1) “classical” pattern of acute uremic state; (2) inflammatory nature of the injured kidneys; (3) modulating effect of AKI of the underlying disease process; and (4) healthcare dilemma. While cytokines/chemokines, leukocyte extravasation, oxidative stress, and certain channel dysregulation are the pathways involving in the remote organ damage. In the current review, we summarized the data from experimental studies to clinical outcome studies in the field of organ crosstalk following AKI. Further, the long-term consequences of distant organ-system, including liver, heart, brain, lung, gut, bone, immune system, and malignancy following AKI with temporary dialysis were reviewed and discussed.

## Introduction

Acute kidney injury (AKI) is a common clinical problem affecting up to 1 % of the general population and 8-15 % of hospitalized patients [1–3], and the incidence is increasing worldwide [4, 5]. Among the critically ill patients, about 50 % develop AKI and 4–15 % have severe AKI needing renal replacement therapy (RRT) support [6, 7]. Besides, AKI was found to be an independent predictor for end-stage renal disease (ESRD) in a large cohort study enrolling 233,803 elderly patients, and the impact of AKI on developing ESRD was even higher than that of previous chronic kidney disease (CKD) (hazard ratio (HR) of AKI alone and CKD alone, comparing with those without AKI or CKD, were 13.0 and 8.4, respectively) [8]. On the other hand, AKI has varied renal recovery rates ranging from 30 % to 70 % in diverse patient types [9].

AKI carries widely-ranged risks of morbidity and mortality in a stepwise manner which increase concurrently with increasing severity and duration of AKI, and even mild temporary AKI is associated with increased morbidity and mortality [8–11]. In addition, the different AKI recovery status might also attribute to mortality and morbidity [9, 10]. Thus AKI has been a global health epidemic problem with soaring incidence, increasing long-term risks for multiple comorbidities and mortality, along with growing healthcare costs [9, 11–13]. Owing to the advancements in preventive and therapeutic strategies, the AKI-associated mortality rates exhibited a significantly declining trend over the decades in both general hospitalized patients [14, 15] and critically ill patients [4]. However, the mortality rates among critically ill patients with AKI remain as high as 40–60 % [7, 16]. Even with survival from the catastrophic AKI events, they sequels of multi-organ damage are disconcerting. One of the possible explanations for this phenomenon is that the RRT, a currently most effective therapy for severe AKI, itself may also carry adverse side effects [17]. Another and perhaps a more important explanation is the distant organ injury, a direct consequence of deleterious systemic effects, following AKI [18, 19]. An increasing body of evidence supports that the extra-renal complications are at least partially responsible for the burden of mortality from AKI [20, 21]. Actually, AKI is often resulted from distant organ injury or systemic illness such as sepsis, and AKI can in turn cause extra-renal organ dysfunction [16, 18]. The term “organ crosstalk” is used to describe “the effects of one dysfunctional organ on the function of another” or “the reciprocal trigger of organ dysfunction of two different organs” [18, 19].

Although the concept of organ crosstalk in AKI is being gradually established, most of the evidence is obtained from animal models [22–24]. The observations in humans are from a limited number of participants in a relatively short follow-up period, [25] or just focusing on the cytokine levels rather than clinical solid outcomes [26]. As such, little is clearly understood on distant organ injury following AKI [18]. The aim of this review is to update the knowledge, from basic aspect to long-term clinical effect, regarding the remote organs consequences following AKI. The organ crosstalk goes from other organs to kidney resulting in AKI will not be covered in this review.

### Distant organ consequences following AKI (Fig. 1)

AKI is gradually considered a pan-metabolic, pan-endocrine and pan-organ problem which exerts negative consequences on many organ systems of the body [27–29]. Briefly speaking, the systemic effects are the reflection of a broad common pathology which ultimately causes an ‘augmented’ inflammation and impairment of immunocompetence during AKI [29, 30]. These multifaceted systemic effects could be categorized into four underlying mechanisms: **(1) “Classical” pattern of acute uremic state** which affects all metabolic and endocrine pathways, causes disruption of electrolyte and volume homeostasis, and further proximate factors have a profound impact on immuno-competence [31–33]. **(2) Inflammatory nature of the injured kidneys.** Animal studies disclosed that injured kidneys may cause obviously higher inflammatory chemokines expression and renal fibrosis [34] as well as profound iron-mediated oxidative stress by disturbing systemic iron homeostasis [35], while supplement with hepcidin may increase the expression of renal H-ferritine and exhibit renal-protective effect [35]. The association between AKI and infection susceptibility is plausible given that acute kidney insults are known to induce changes in gene regulation, oxidative stress, inflammation, leukocyte trafficking, and apoptosis and to incite systemic and distal organ injury. The inflammatory process might eventually transform into systemic inflammatory reaction mediating distant organ injury [24, 36]. **(3) Modulating effect of AKI of the underlying disease process.** A great modulating effect on the underlying disease process of other organ-systems would be induced by the disturbed cytokine/chemokine homeostasis in AKI, which may be attributable to the decreased renal clearance and/or increased production of these cytokine/chemokine [34, 35, 37–39]. **(4) Healthcare dilemman** RRT support is generally considered as necessary for AKI patients with profound biochemical disarrangement or fluid overload [40]. However, RRT itself is proven to carry significant risks for adverse patient outcome owing to the hemodynamic instability and nutrients loss during RRT, as well as reactive oxygen species and inflammatory reaction secondary to the bio-incompatibility of artificial kidney, and the use of unfractionated heparin [17, 30, 41, 42]. The adequate serum level of antibiotics and anticoagulant are also difficult to achieve and maintain in AKI patients with RRT [17], which may disturb infection control or cause complications. Besides, the use of mechanical ventilation and hemodialysis catheters also raise the risk of complications.

**Fig. 1:**
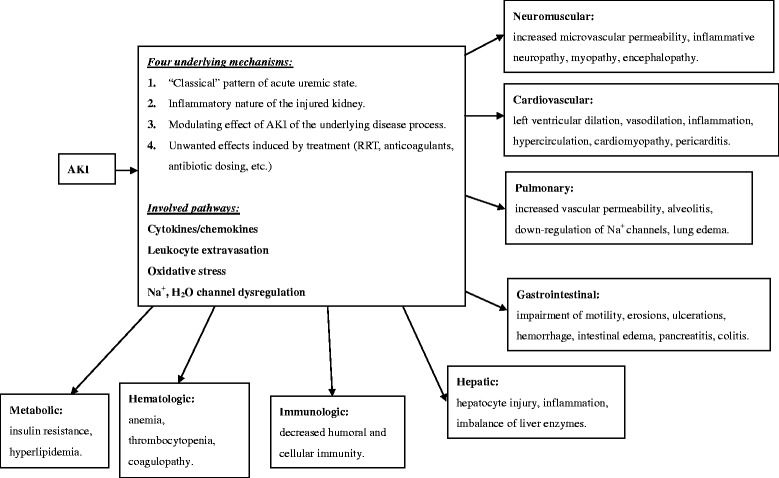
Proposed mechanism of distant organ consequences following AKI [17, 18, 21, 24, 30–42]. Abbreviations: AKI, acute kidney injury; RRT, renal replacement therapy

Based on the aforementioned mechanisms, several complex pathways are involved in the crosstalk of organs during AKI, causing injury in distant organ-systems including neuromuscular, cardiovascular, pulmonary, gastrointestinal, hepatobiliary, immunologic, hematologic, and metabolic systems [18, 19, 21, 30, 39].

### Long-term remote organ consequences following AKI with temporary dialysis

This review focused on the long-term pivotal effects of *de novo* RRT-requiring AKI on extra-renal organ systems. To avoid the confounding detrimental effects of chronic RRT, which has been proven to result in worse patient outcomes, the following review only covered studies in which the enrolled subjects were limited to those recovered from the most severe form of AKI, i.e. those with transient RRT-requiring AKI (Table 1).

**Table 1:** Long-term remote organ consequence following AKI undergoing RRT

Outcomes	Hazard ratio (95 % Confidence interval)			References and details
	No-AKI	Recovery-AKI	Non-recovery AKI	
Coronary event	Reference	1.67 (1.36–2.04)^a^	–	Wu et al. [12]; n = 4,869x2^b^; mean f/u: 3.31 yrs
Upper gastrointestinal bleeding	Reference	1.30 (1.14–1.48)^a^	ESRD after AKI recovery 2.31 (1.92–2.79)^a^	Wu et al. [87]; n–4,565x2^b^; median f/u: 2.33 yrs
Incident Stroke	Reference	1.25 (1.10–1.65)^a^	–	Wu et al. [60]; n = 4,315x2^b^; median f/u: 3.36 yrs
Severe sepsis	Reference	1.58 (1.15–2.16)^a^	ESRD after AKI recovery 1.99 (1.71–2.31)^a^	Lai et al. [103]; n = 2,983 + 11,932^b^; median f/u: 3.96 yrs
Active tuberculosis	Reference	3.84 (2.07–7.10)^a^	6.39 (3.57–11.45)^a^	Wu et al. [68]; n = 2,909 + 11,636^b^; mean f/u: 3.6 yrs
Malignancy	0.66 (0.45–0.98)^c^	Reference	1.49 (1.02–2.03)^c^	Chao et al. [100]; n = 623x3^b^; mean f/u: 3.7 yrs
Bone fracture	Reference	6.59 (2.45–17.73)^a^	–	Wang et al. [92]; n = 448 + 1,792^b^; mean f/u: 3.9 yrs.

Data are represented as Hazard Ratio (95 % Confidence Interval)

All the studies defined renal recovery by independence from RRT are population-based study based on Taiwan National Health Insurance Research Database. AKI defined by RRT initiation, while recovery defined by withdraw from RRT

Abbrevations: *AKI* acute kidney injury, *ESRD* end-stage renal disease, *f/u* follow up

^a^Statistical significancy comparing with no-AKI group

^b^Matched patients

^c^Statistical significancy comparing with recovery-AKI group

^d^Statistical significancy comparing with non-recoveryAKI group

#### Kidney-heart crosstalk

Most patients with acute decompensated heart failure have underlying renal insufficiency, which, alone or along with acute renal insults, expose the patients to adverse clinical outcomes [43–45]. The effects of cardiac failure on kidney injury may be through hemodynamic-, humoral- and immune-mediated pathways [46–49]. Reversely, AKI may also result in acute cardiac disorder via some mechanisms such as: (1) increased preload secondary to AKI-induced salt and water retention; (2) myocardial damage due to neutrophil trafficking, myocyte apoptosis, endothelial dysfunction, as well as elevated level of inflammatory cytokines (interleukin (IL)-1, IL-6, and tumor necrosis factor (TNF)-α) resulting from increased production and impaired clearance [21, 50–52].

To identify the association of AKI with long-term cardiovascular risk, Wu et al. [12] compared 4,869 patients who recovered from *de novo* RRT-requiring AKI (recovery-AKI group) and the same number of matched patients (no-AKI group). To make an unbiased estimate of the confounders, propensity score method was used to adjust baseline characteristics which might affect dialysis withdrawal and subsequent patient outcomes. The control group (no-AKI group) was matched with the exposure group (recovery-AKI group) on the basis of age, sex, same calendar year of index hospitalization, and comorbidities before and during index hospitalization. Furthermore, the results were further validated by analysis of a prospectively constructed database.

With a mean follow-up period of 3.31 years, the patients in “recovery-AKI group” had 1.7 times higher risk of coronary events than “no-AKI group”, independent of the effects of subsequent progression to CKD and ESRD. In an adjusted comparison, “RRT-requiring AKI alone” (3.3 times hazard) was associated with even higher long-term coronary events than “diabetes alone” (2.8 times hazard). Furthermore, adjusting for interim coronary events attenuated the association between RRT-requiring AKI and subsequent risk of death, supporting the hypothesis that coronary events are in the causal pathway linking AKI and mortality. The study substantiated the interaction of cardiorenal syndrome type 3, which describes impaired myocardial function by various interconnected pathways in AKI [53]. The association between AKI and subsequent risk for cardiac events were also identified in some other studies [54, 55].

#### Kidney-brain crosstalk

AKI has neurological complications including dizziness, attention deficits, tremor, seizure, altered mental status, delirium, and even death [21]. Cellular and soluble inflammatory mediators as well as uremic toxins contribute to the neurological symptoms. Animal studies using mice found that AKI may subsequently result in increased vascular permeability, disruption in the blood-brain barrier, increased cerebral proinflammatory cytokines (IL-6, IL-1β, IL-12, keratinocyte-derived chemokine, granulocyte-colony stimulating factor, and glial fibrillary acidic protein), and increased neuronal pyknosis and microgliosis (up-regulation of brain macrophages) [21, 23]. Microgliosis is a hallmark of cerebral inflammation and is implicated in the pathology of neurodegenerative diseases [56]. Alterations in calcium concentrations, water handling and neurotransmitter turnover may also play roles in functional changes in the brain after AKI [21]. Additionally, posterior reversible encephalopathy syndrome (PRES) has been reported in AKI patients with [57] and without hypertension [58]. Hypertension that exceeds the limits of auto-regulation of the brain, immune response and endothelial dysfunction may account for the development of PRES [58]. Reversely, although about one-fourth of hospitalized patients with acute stroke develop AKI, the renal changes secondary to neurological illness is less established [21]. Increased inflammation is observed in renal allografts from brain-dead donors [59].

A study determining the long-term effect of AKI on *de novo* stroke was carried out with a median follow-up period of 3.36 years [60]. The patients in “recovery-AKI group” (n = 4,315) had 1.3 times elevated risk of developing incident ischemic and hemorrhagic stroke than those in “no-AKI group” (n = 4,315) regardless of progression to subsequent CKD. The risk factors for kidney injury leading to an AKI event may persist and eventually lead to future stroke without a direct causal association with preexisting CKD. AKI may thus amplify the long-term risk of incident stroke and mortality, and the impact is similar to diabetes.

Over the 3 years of follow-up, RRT-requiring AKI is associated with an increased risk of dementia after critical illness [61]. Other independent risk factors include infection, severe sepsis [61], and a prolonged duration of delirium [62]. In this regard, interventions directed at reducing delirium and preserving renal function may mitigate brain injury associated with critical illness.

#### Kidney-immune system crosstalk

The profound impact on immunocompetence is one of the most important complications of AKI since kidney is an important immunologic organ. AKI can cause short- and long-term, either renal or systemic, immune-modulation. First of all, both T cell population and phenotype alteration within and outside the kidney were observed in animal model after ischemic/reperfusion kidney injury [63, 64], and these changes can last for about three months. Moreover, either ischemia/reperfusion injury itself or protein binding uremic toxin can leads to epigenetic modifications resulting in distinct genomic signatures [36, 65, 66]. In concordance to these findings, our group had also observed an increase in long-term risk of severe sepsis and tuberculosis after RRT-requiring AKI. The adjusted risk of developing severe sepsis prompting hospitalization was approximately two-fold higher among AKI survivors compared to non-AKI patients (incidence rate of 6.84 versus 2.32 per 100 person-years) [67]. Patients who recover from RRT-requiring AKI also had significantly higher incidence of TB than patients without AKI (HR, 3.84; p, 0.001) [68]. Taken together, studies from bench to bedside all showed that AKI alters host immunocompetence even after renal function recovery, and these alterations could affect patient’s long-term outcome.

On the other hand, several studies have shown that certain cytokine or chemokine gene polymorphism are related to the occurrence or severity of AKI. In clinical studies, TNF-α and IL-10 phenotype predict AKI and mortality in hospitalized patients [69, 70], while transforming growth factor (TGF)-*β* and interferon-*γ* genotype do not [71]. C-C chemokine receptor 5 knock-out mice showed increased susceptibility to lipopolysaccharide‑induced acute renal injury [72]. It is therefore reasonable to postulate that certain genetic backgrounds would affect the susceptibility to immune derangement, inflammation, sepsis and AKI, whichever happens first.

#### Kidney-lung crosstalk

Respiratory consequences are the most clinically relevant distant organ injury in AKI, and AKI is also commonly seen in patients with pulmonary inflammation and mechanical ventilation [21, 30]. AKI alters peripheral vascular responses by increasing oxidative stress, likely in the endothelium [73]. As to renal cytokine production, complement activation, toll-like receptor-2, and toll-like receptor 4 signaling contribute to cytokine production after AKI [74, 75]. Experimental studies demonstrated that AKI results in lung injury via following pathways: (1) lung edema, which may be resulted from increased pulmonary vascular permeability and dysregulated ion transport channels; (2) increased cytokines and chemokines related to impaired renal clearance and increased production; and (3) increased leukocyte trafficking with mononuclear phagocyte production. Besides, AKI may express modulatory effects that vary with the severity of lung injury [21, 76].

Following AKI, serum IL-6 increases in the absence of a counter anti-inflammatory response by spleen, and it brings an exuberant proinflammatory response and mediates lung injury via Chemokine (C-X-C motif) ligand 1 production in mice [77, 78]. In clinical studies, inflammatory cytokines, such as IL-6 and/or IL-8, are potential mediators of the distant organ damage from AKI. Serum IL-6 correlates with endothelial dysfunction in human [79], while elevated levels of IL-6 are associated with prolonged ventilator weaning times and increased mortality in patients with AKI and acute lung injury [80].

A study evaluating the long-term risk and outcome of active tuberculosis after AKI was conducted enrolling 2,909 RRT-requiring AKI patients (AKI-group) and 11,636 control individuals (no-AKI group) with a mean follow-up period of 3.6 years [68]. The “AKI-group” had 7.7 times higher risk of active tuberculosis than general population. Comparing with “no-AKI group”, the “recovery-AKI group” had 3.8 times, and “non-recovery AKI group” had 6.4 times elevated risk of getting tuberculosis. Besides, active tuberculosis was associated with 1.3 fold increased risk of long-term all-cause mortality after RRT-requiring AKI. These results raise concerns that the increasing global burden of AKI would also increase the incidence of active tuberculosis. The increased long-term risks for severe sepsis and tuberculosis might be the results of impaired pulmonary and immunologic function implicated by AKI.

#### Kidney-gut crosstalk

Gut is a newly-found organ which would be remotely injured during AKI. The inflammatory response and hypervolemia related to AKI alter the permeability of mesenteric vascular bed and promote the formation of intestinal edema, which is a hallmark of intestinal failure in sepsis [81]. Gut has been known as an amplifier of systemic inflammatory response syndrome in the setting of shock, gut hypoperfusion [82] and intestinal edema [81] through the mechanisms including increased intestinal permeability, disruption of mucosal integrity, liberation of proinflammatory mediators, translocation of intestinal microorganisms and resultant endotoxinemia. The augmented systemic inflammatory reaction by gut would in turn aggravate AKI, giving rise to a vicious cycle.

Malnutrition and calorie deficit are associated with poor renal outcome and mortality in AKI patients. The condition is moreover complicated by changes in gut mobility, insulin resistance, and hypercatabolic state due to inflammatory mediators and neuroendocrine derangement [83]. Despite the lack of large-scale randomized controlled trials in the AKI population, early enteral nutrition should be used to preserve gut function and possibly to prevent stress ulcer hemorrhage [84, 85]. A recent multicenter, randomized controlled trial showed that early parenteral nutrition to supplement insufficient enteral nutrition does not increase the incidence of AKI, but prolongs the duration of renal replacement therapy [86]. Nutritional requirements should be individualized and frequently reassessed to avoid under- or overfeeding, azotemia, hyperglycemia, hypertriglyceridemia, electrolyte and acid-base imbalance, and fluid overload [85].

After a median follow-up period of 2.33 years, Wu et al. [87] disclosed that “recovery-AKI” was an independent predictor of long-term upper gastrointestinal bleeding, and that upper gastrointestinal bleeding was a significant risk factor of long-term mortality. The reported incidence of upper gastrointestinal hemorrhage following transient RRT-requiring AKI was about one hundred times greater than that of the general population. Peptic ulcers accounted for two-thirds of upper gastrointestinal bleeding episodes following AKI. Although AKI incites inflammation, an “immunoparalysis” state, similar to that ensuing from sepsis and critical illness [88, 89], may follow and render the effects of *Helicobacter pylori* more pernicious. The long-term incidence of upper gastrointestinal bleeding in patients with “recovery-AKI” (15–20 %) outnumbers the short-term incidence in critically ill patients (1–6 %) [90], suggesting the impact of AKI on the microvascular injury [91] might last long, although a compelling mechanism remains obscure. The high accumulation rates of nonsteroidal anti-inflammatory drugs and anti-platelets use after AKI in this cohort further augmented the possibility of gastrointestinal bleeding. Physicians should be more prudent about the use of ulcerogenic agents in this patient population.

#### Kidney-bone crosstalk

Wang et al. [92], by using a nationwide population-based cohort study, evaluated the associations between AKI and long-term risk on bone fractures. After a series of selecting and matching process, a “recovery-AKI group” containing 448 patients who developed transient RRT-requiring AKI for less than 90 days and who didn’t have diagnosis of bone fracture, and a “no-AKI group” containing 1,792 propensity score-matched individuals (with 1:4 ratio) without AKI or RRT were identified. Comparing with the “no-AKI group”, those in “recovery-AKI group” had 6.5 folds higher risk of developing bone fracture in a mean follow-up period of 3.9 years. Even after adjustment with other covariates, AKI is still independently associated with higher risk of bone fracture irrespective of subsequent development of ESRD. Long‐term bone fractures may also negatively impact patient mortality.

AKI is thought as a renowned predecessor of CKD [93] because the pathological changes of renal osteodystrophy occur earlier than previously expected CKD stages [94], and dysregulated mineral hormones also occur in AKI. It is plausible that earlier changes in vitamin D metabolites and/or fibroblast growth factor (FGF)-23 levels during AKI are responsible for the subsequent bone structural abnormalities [92]. Besides, some risk factors identified to predict bone fracture in the study, such as peripheral vascular disease and neurologic problems, are also at least partly related to AKI. The results from the study additionally offer an insight into the impact of bone disease [92].

#### Kidney-hepatic crosstalk

Though the clinical evidence of the association between acute liver injury and AKI is well documented, the mechanisms and pathways between kidney-hepatic crosstalk remains to be investigated [95]. An experimental study showed that hepatic ischemic/reperfusion injury (HIRI) abrupt increases multiple cytoprotective proteins, such as neutrophil gelatinase-associated lipocalin (NGAL), heme oxygenase-1 and hepcidin, inducing a renal cortical “stress response” [96]. Though modest azotemia occurs, the HIRI-induced azotemia appears to a pre-renal state rather than an intrinsic renal damage. Thus, despite clinical evidence of acute liver injury predispose to AKI, it seems plausible that AKI contributes to subsequent liver damage more directly. Growing evidence showed that AKI has significant effect on liver inflammatory response, as well as drug or other nutrient metabolism, and even patient outcomes [19]. Experimental studies showed that AKI results in increased vascular permeability, neutrophil and T-lymphocyte infiltration in the liver [18]. Besides, AKI activates oxidative stress, decreases antioxidants level and upregulate the expression of injury-promoting molecules, leading to apoptosis and tissue damage of hepatocytes [30, 97]. Among the related cytokines, IL-6 is a well-known one induced in AKI and could activate Kuffer cell to further produce other inflammatory cytokines including IL-10 [98]. As to the results of clinical studies, an analysis from a randomized control study found that the occurrence of a subsequent AKI would increase mortality rate from 28 % to 58 % among patients with acute liver injury [99].

#### Kidney-malignancy

A nationwide population study using 1,000,000 representative database during 2000–2008 was conducted by Chao et al. [100] who identified 623 individuals recovering from RRT-requiring AKI (recovery-AKI group) and 623 patients developing ESRD during follow-up (non-recovery AKI group), along with an age, sex, and diabetes status-matched control group (non-AKI group). After a mean follow-up period of 3.7 years, the standardized incidence ratios of all cancers are higher in both recovery-AKI group (1.21) and non-recovery AKI group (1.31) when using general population as reference. And the incidences of *de novo* malignancy were gradually increased from “no-AKI group”, “recovery-AKI group”, to “non-recovery AKI group” (2.6, 2.9, and 4.2 per 100 person-year, respectively). Overall speaking digestive tract (4.9 %), Genitourinary tract (3.0 %), and respiratory tract (1.7 %) were the top three sites of *de novo* cancers occurrence. Comparing with the “recovery-AKI group”, the “no-AKI group” had lower risk (HR, 0.66) while the “non-recovery AKI group” had higher risk (HR, 1.44) of developing malignancy.

This study demonstrates patients with non-recovery AKI developed more genitourinary cancers (5.1 %), while recovery patients developed more respiratory tract cancers (2.2 %) in the long run. The relationship presumably includes viral carcinogenesis, uremic immune suppression, and toxin exposure. The acute or chronic inflammation and the ensuing regeneration processes might carry the possibility of promoting the subsequent uncontrollable proliferation and neoplasm formation [50]. The increase in protein binding uremic toxin during AKI also could lead to epigenetic modification and potentially increases chances of tumor-suppressor gene silencing [66, 101]. This study shows patients requiring even temporary RRT have higher long-term risk of developing cancers, independent of subsequent progression to CKD and ESRD.

## Discussion

In the current review, the development of severe AKI requiring RRT is found to have significant impact on long-term morbidities of distant organs. These epidemiological findings in large-scaled population studies echo and add much strength to the proposed underlying mechanisms and involved pathways in organ crosstalk of AKI. Besides, Pickering et al. [102] evaluated trial outcomes using mathematic modeling of serum creatinine (SCr) changes in AKI, and found that the “time of SCr elevation” (duration of AKI) is associated with an efficient outcome-predictive power. These could explain the impact of recovery status on patient outcomes. From the nationwide population studies comparing the effects from AKI with different recovery status (recovery versus non-recovery AKI) [68, 87, 100, 103], the “AKI duration”-dependent effect of long-term distant organ injury from RRT-requiring AKI represent its real impact.

Similar results were also revealed among patients with less severe form of AKI not requiring RRT support, no matter the study focused on diabetic population [104] or not [105]. The findings indicated that the AKI *per se*, beside RRT, speaks for itself. Recently, a retrospective multicenter study enrolling 447 critically-ill patients from six intensive care units evaluated the impact of duration (transient and persistent AKI) and severity of AKI (stage 3 AKI) on patient outcomes [106]. After adjustment with all confounding factors, “persistent AKI” was found as an independent factor associated with worse hospital survival. However, when “stage 3 AKI” was put into the final multivariate medel, “stage 3 AKI” replaced “persistent AKI” as an independent factor with lower survival. The findings are interesting and may be interpreted that “severity of AKI” is more relevant than “duration of AKI” in influencing patient prognosis.

An episode of AKI is proven to result in prolonged impairment of renal blood flow and clearance even the SCr has apparently returned to baseline [107]. Maladaptive repair after AKI, which is characterized by persistent parenchymal inflammation with increased numbers of myofibroblasts and accumulation of extracellular matrix, may lead to CKD. And the risk factors for the maladaptive repair response include the type and duration of injury [108].

The exact causative association between AKI and the long-term distant organ injury risks is still not clearly recognized [109]. Nonetheless, when the risk factors that had engendered an AKI event may persist [110] and concomitantly or subsequently lead to distant organ damage without direct causal association between the past CKD. Cytokine surge during AKI is at least partly resulted from subsequent impaired filtration and clearance of uremic toxins, and its impact is thought to attribute to the remote damage between kidney and distant organs [18]. Although it is seemingly intuitive that the duration of AKI would be positively associated with remote organ damage, the direct evidence addressing this is lacking.

The findings from aforementioned studies raise the possibility that AKI might trigger a cascade of perturbations which are not completely resolved. And certain non-traditional risk factors, such as impaired endothelial progenitor cells, endothelial dysfunction, inflammatory response, oxidative stress, hyper-homocysteinemia, and thrombogenic factors during AKI, are involved in the he pathogenic mechanisms [111, 112]. These risk factors may play a role in accelerated atherosclerosis in the arteries of both the kidney and remote organs, making AKI a non-modifiable entity. Besides, the RRT hemofiltrate from AKI patients inhibited *in vitro* neutrophil chemotaxis, oxidative metabolism, and apoptosis, which could favorably affect endothelial evolution [113]. Taken together, AKI, in addition to the traditional cardiovascular equivalent, serves as a “kidney specific” risk factor associated with distant organ injury.

Since AKI is known as a contributing factor to late-stage CKD, it is possible that early kidney changes involving endothelial phenotypic transition may have already taken place during acute events, paving the way toward progressive renal function deterioration. Recently, FGF-23, a novel regulator of mineral metabolism which is markedly elevated in AKI [114], is regarded as an index of subclinical cardiovascular pathology and associated with adverse cardiovascular outcome [115]. Likewise, the expression of NGAL, a marker of renal tubular injury, is also related to increased cardiovascular and all-cause mortality independent of traditional cardiovascular risk factors [116].

Besides the concept of organ crosstalk during AKI that AKI itself could cause a number of systemic vascular endothelial alterations which impact cardiovascular health [109], some possible explanations for the remote organ injury from recoverable AKI are summarized below. First, AKI may serve as a surrogate of other end-organ damages. A recoverable AKI may be a less severe AKI event which itself does not cause damage but merely reflects a sicker patient population who have less renal reserve and more subsequent medical complications. Second, regardless renal recovery status, AKI patients had higher probability to be hospitalized for a longer duration than those without AKI, thus may get higher risks of medical complications [105].

Among the managements of AKI-related remote organ consequences, early identifying and stratifying patient in risk, as well as preventive measures to avoid the occurrence and evolution of AKI are the most important strategy. Over the past decade, many novel biomarkers with important biological function in the pathogenesis of AKI were identified and validated. Generally speaking, the biomarkers of AKI are categorized into three groups, namely, functional biomarkers (such as SCr, cystatin C, α1- or β2-microglobulin), tubular enzymes (such as α-glutathione-S-transferase (α-GST)), and unregulated proteins (such as NGAL, L-type fatty acid-binding protein (L-FABP), kidney injury molecule-1 (KIM-1) and IL-18) [117].

A rapidly elevated urine concentration of NGAL, L-FABP, α-GST, KIM-1, and IL-18 is indicative of AKI, achieving an early detection of AKI about 24 to 48 hours before the rise of SCr level [118]. According to the varied characteristic of individual biomarker along with the concentration change in serum and urine sample, the biomarkers are also valuable in making differential diagnosis of AKI among the critically ill patients [119]. Besides, the elevated level of some biomarkers are of important prognostic value. An early assessment of prognosis may result in better outcomes [119].

Since cytokines and chemokines were thought to play important roles in the organ crosstalk in AKI [18, 19, 21, 30, 39], it is reasonable to consider “removing cytokines/chemokines” as a potential management strategy to preventing remote organ damage following AKI. Although hemofiltration could remove inflammatory cytokines, it does not affect patient mortality rates regardless the amount of applied dosage among the patients with septic shock in which inflammatory mediators contribute to patients outcomes [120]. Actually, standard dialysis membrane could only remove limited amount of cytokines even with high volume hemofiltration [121]. A kind of recently-developed dialysis membranes with moderately larger pore size, called high cutoff membranes, is found to have better effectiveness in removing cytokines than standard membranes and associated with better immunologic function and survival in experimental models of sepsis [17, 122]. Besides, “coupled plasma filtration and adsorption” is found to improve function of immunity and circulating leukocytes in septic shock [123], while “polymycin B hemoperfusion” is suggestive of beneficial effect on arterial pressure, gas exchange, and mortality [124]. However, the evidence exactly focusing on the association between RRT and remote organ consequence is lacking. Further large-scaled, prospective study is warranted to evaluated the association among RRT intervention, cytokines/chemokines/oxidative stress, and remote organs injury, in AKI patients undergoing optimized RRT in a way that the multiple negative effects of the acute uremic state are mitigated.

## Conclusion

In conclusion, an increasing body of research demonstrated the organ crosstalk during AKI has significant impact on long-term distant organ-system comorbidities, the impact persist despite subsequent renal function recovery. Recognizing key biomarkers of inflammation as novel therapeutic targets may improve the quality of diagnosis and therapy since cytokines and chemokines play important roles in the distant organ injury during AKI. Besides, optimizing RRT for remove certain cytokines, as well as decreasing inflammatory and acute uremic status may probably be a useful strategy to avoid distant organ injury.

## Abbreviations

AKI: acute kidney injury
α-GST: α-glutathione-S-transferase
CKD: chronic kidney disease
ESRD: end-stage renal disease
FGF: fibroblast growth factor
HIRI: hepatic ischemic/reperfusion injury
HR: hazard ratio
IL: interleukin
KIM-1: kidney injury molecule-1
L-FABP: L-type fatty acid-binding protein
NGAL: neutrophil gelatinase-associated lipocalin
PRES: posterior reversible encephalopathy syndrome
RRT: renal replacement therapy
SCr: serum creatinine
TGF: transforming growth factor
